# Pharmacological inhibition of GSK-3 in a guinea pig model of LPS-induced pulmonary inflammation: II. Effects on skeletal muscle atrophy

**DOI:** 10.1186/1465-9921-14-117

**Published:** 2013-11-01

**Authors:** Koen JP Verhees, Nicholas AM Pansters, Hoeke A Baarsma, Alexander HV Remels, Astrid Haegens, Chiel C de Theije, Annemie MWJ Schols, Reinoud Gosens, Ramon CJ Langen

**Affiliations:** 1 Department of Respiratory Medicine, School for Nutrition, Toxicology and Metabolism (NUTRIM), Maastricht University Medical Centre + (MUMC+), PO box 5800, 6202, AZ Maastricht, The Netherlands; 2 Department of Molecular Pharmacology, University of Groningen, Groningen, The Netherlands; 3 Groningen Research Institute for Asthma and COPD, University of Groningen, Groningen, The Netherlands

**Keywords:** COPD, Inflammation, LPS, Skeletal muscle atrophy, Myogenesis

## Abstract

**Background:**

Chronic obstructive pulmonary disease (COPD) is accompanied by pulmonary inflammation and associated with extra-pulmonary manifestations, including skeletal muscle atrophy. Glycogen synthase kinase-3 (GSK-3) has been implicated in the regulation of muscle protein- and myonuclear turnover; two crucial processes that determine muscle mass. In the present study we investigated the effect of the selective GSK-3 inhibitor SB216763 on muscle mass in a guinea pig model of lipopolysaccharide (LPS)-induced pulmonary inflammation-associated muscle atrophy.

**Methods:**

Guinea pigs were pretreated with either intranasally instilled SB216763 or corresponding vehicle prior to each LPS/saline challenge twice weekly. Pulmonary inflammation was confirmed and indices of muscle mass were determined after 12 weeks. Additionally, cultured skeletal muscle cells were incubated with tumor necrosis factor α (TNF-α) or glucocorticoids (GCs) to model the systemic effects of pulmonary inflammation on myogenesis, in the presence or absence of GSK-3 inhibitors.

**Results:**

Repeated LPS instillation induced muscle atrophy based on muscle weight and muscle fiber cross sectional area. Intriguingly, GSK-3 inhibition using SB216763 prevented the LPS-induced muscle mass decreases and myofiber atrophy. Indices of protein turnover signaling were unaltered in guinea pig muscle. Interestingly, inhibition of myogenesis of cultured muscle cells by TNF-α or synthetic GCs was prevented by GSK-3 inhibitors.

**Conclusions:**

In a guinea pig model of LPS-induced pulmonary inflammation, GSK-3 inhibition prevents skeletal muscle atrophy without affecting pulmonary inflammation. Resistance to inflammation- or GC-induced impairment of myogenic differentiation, imposed by GSK-3 inhibition, suggests that sustained myogenesis may contribute to muscle mass maintenance despite persistent pulmonary inflammation. Collectively, these results warrant further exploration of GSK-3 as a potential novel drug target to prevent or reverse muscle wasting in COPD.

## Background

Chronic obstructive pulmonary disease (COPD) is characterized by an irreversible and persistent airflow limitation and is associated with pulmonary inflammation [1-3]. COPD is also typified by significant extra-pulmonary manifestations, that contribute to increased morbidity and mortality, independent of the primary pathology [4]. Interestingly, pulmonary inflammation has been suggested as a trigger and perpetuating factor in the local and systemic pathology of COPD. One of the major systemic consequences of COPD is peripheral muscle dysfunction, comprising a loss of muscle strength and endurance, respectively [5-7]. A major cause of loss of muscle strength is the decrease in muscle mass due to myofiber atrophy [8-11].

Skeletal muscle atrophy or muscle wasting may be the consequence of a disturbed balance between protein synthesis and degradation in favor of the latter; due to either accelerated breakdown of muscle proteins, or reduced protein synthesis [9,12,13]. Insulin-like growth factor I (IGF-I) and insulin are both anabolic factors that affect cellular protein turnover via a well-characterized signaling conduit that includes phosphorylation of phosphatidylinositol-3 kinase (PI-3K), resulting in the activation of Akt/PKB (hereafter termed Akt) [13]. Phosphorylated Akt can, in turn, stimulate protein synthesis by activating mammalian target of rapamycin (mTOR) signaling, characterized by phosphorylation of its downstream substrates 4E-BP1 and p70S6K [13]. Conversely, Akt activation results in the phosphorylation and subsequent cytoplasmic retention of the Forkhead box O (FoXO) class of transcription factors, which have been implicated in the coordination of proteolytic gene expression [14-16].

In addition to protein turnover, myonuclear turnover, i.e. the balance between myonuclear loss and myonuclear accretion, may constitute an additional cellular mechanism determining muscle mass [17]. Efficient regeneration and restoration of muscle mass following injury or recovery from atrophy requires activation, proliferation and subsequent differentiation of satellite cells into myoblasts that fuse with existing or form new myofibers [18,19]. Besides myoblast fusion, myogenic differentiation is characterized by increased transcriptional activity of muscle regulatory factors (MRFs) (e.g. MyoD, myogenin), which promote the expression of muscle-specific genes, including contractile/sarcomeric proteins such as troponin-I (TnI), myosin light chain (MyLC) and myosin heavy chain (MyHC), and enzymes involved in muscle energy metabolism (e.g. muscle creatine kinase (MCK)) [20].

Besides the pulmonary pathology, systemic inflammation in COPD, which manifests itself as increased activation of circulating inflammatory cells and elevated levels of TNF-α or IL-1β, as well as increased serum concentrations of acute phase proteins such as C-reactive protein (CRP) [21-23], may directly or indirectly contribute to skeletal muscle atrophy [24,25]. In a mouse model of pulmonary inflammation, we recently demonstrated that muscle NF-κB activation was required for the transition from inflammatory- to muscle atrophy signaling [26], suggesting that systemic inflammation contributes to the loss of skeletal muscle mass following acute pulmonary inflammation. Furthermore, the release of glucocorticoids (GCs) as an endogenous response to inflammation, or the administration of synthetic GCs to COPD patients as a common intervention during acute exacerbations or end stage disease may also evoke or aggravate muscle wasting as GCs are potent inducers of muscle atrophy [27-30].

Currently, pharmacological treatment approaches of muscle atrophy in COPD are limited [31-33], and therapeutic interventions should be aimed at suppression of triggers of muscle atrophy, e.g. pulmonary inflammation, or at direct modulation of the signaling pathways that regulate muscle mass. Glycogen synthase kinase-3 (GSK-3) is a ubiquitously expressed serine/threonine kinase, occurring in two closely related isoforms, namely GSK-3α and GSK-3β, which share extensive homology in their kinase domains [34-36]. GSK-3β is a signaling protein directly downstream of Akt, which plays an important role in a myriad of cellular processes, including inflammatory signaling [37,38] and protein synthesis [39], through regulation of mRNA translation initiation via suppression of eIF2B activity. Recent data from our group and others suggests a pivotal role for GSK-3β in the determination of muscle mass, as it is involved in both protein and myonuclear turnover. Concretely, it was established that muscle atrophy, resulting from increased proteolysis signaling following synthetic GC-treatment, requires GSK-3β [40]. In another study by our group physiological and pharmacological GSK-3 inhibition enhanced myoblast fusion and myotube formation, in support of an important role of GSK-3 in the regulation of myonuclear turnover [41].

Considering the significance of GSK-3 in the cellular processes controlling inflammatory signaling and muscle mass, the purpose of this study was to assess the potential therapeutic effects of GSK-3 enzyme inhibition on muscle wasting in an established guinea pig model of lipopolysaccharide (LPS)-induced pulmonary inflammation, using the selective inhibitor 3-(2,4-dichlorophenyl)-4-(1-methyl-1H-indol-3-yl)-1H-pyrrole-2,5-dione (SB216763) [42]. The data presented in this study demonstrate that topical application of a GSK-3 inhibitor does not affect pulmonary inflammation, but reduces skeletal muscle atrophy. Subsequent cell culture experiments suggested this may involve maintenance of myogenesis, as GSK-3 inhibition restored muscle differentiation in the presence of effectors of systemic inflammation. Collectively, these current findings warrant further exploration of GSK-3 as a novel therapeutic target in the treatment of skeletal muscle atrophy in COPD.

## Methods

### Animals

Outbred, male, specified pathogen-free Dunkin Hartley guinea pigs (Harlan, Heathfield, UK) were used in this study. All protocols described in this manuscript were approved by the University of Groningen Committee for Animal Experimentation.

### Experimental protocol

Thirty-six guinea pigs, 12 ± 4 wks of age were randomly assigned to four experimental groups (n = 9), namely: (1) vehicle-treated, saline-challenged; (2) SB216763-treated saline-challenged; (3) vehicle-treated, LPS-challenged, and (4) SB216763-treated, LPS-challenged. The guinea pigs were treated twice per week for 12 consecutive weeks by intranasal instillation of 100 μl SB216763 (sterile, 2 mM in 10% (v/v) DMSO in saline) or vehicle (100 μl 10% (v/v) DMSO in sterile saline). After the intranasally instilled solution was aspirated, the animals were kept in an upright position for an additional 2 min, to allow sufficient spreading of the fluid throughout the lungs. The animals were intranasally instilled with 100 μl LPS (10 mg/ml in sterile saline) or sterile saline, 30 min *post* SB216763 or vehicle instillation. SB216763 is a selective GSK-3 inhibitor (3-(2,4-dichlorophenyl)-4-(1-methyl-1H-indol-3-yl)-1H-pyrrole-2,5-dione) (Tocris Cookson, Bristol, UK) and the LPS was derived from *Escherichia coli*, serotype 055:B5 (Sigma-Aldrich, MO, USA). Twenty-four hours after the last instillation, the guinea pigs were sacrificed by experimental concussion, followed by rapid exsanguination. Next, the lungs and a series of hind limb muscles including the M. gastrocnemius, M. tibialis anterior, M. plantaris and M. extensor digitorum longus (EDL) were collected using standardized dissection methods. Independent muscle weights of a single hind limb were measured and all tissues were immediately flash-frozen in liquid nitrogen.

### Tissue processing and histological analyses

The EDL muscles were embedded in Tissue-Tek (Sakura Finetek, the Netherlands) and sectioned on a Leica CM3050 S cryostat at −20°C. Subsequently, serial cross-sections (5 μm) were stained with the following primary antibodies: anti-Type I MyHC (#A4840) (Developmental Studies Hybridoma Bank, Iowa City, IA, USA), and anti-laminin (#L-9393) (Sigma-Aldrich) to determine the fiber cross-sectional area (CSA) and fiber type distribution. The sections were incubated with the following secondary antibodies: goat anti-mouse IgM Alexa Fluor 555 (#A-21426) and goat anti-rabbit IgG Alexa Fluor 350 (#A-11069) (both from Invitrogen, CA, USA). Digital images of the stained sections were taken under 200X total magnification using an Eclipse E800 microscope (Nikon, Japan) connected to a digital camera (DXM, 1200 NF, Nikon, Japan). The CSA was measured after having identified five non-overlapping regions containing a total of 100–200 individual fibers per animal, which were then analyzed using Lucia Software (version 4.81).

### Cell culture

The murine skeletal muscle cell line C_2_C_12_ (ATCC # CRL1772) was cultured in growth medium (GM), composed of low glucose (1 g/l) Dulbecco’s Modified Eagle Medium (DMEM) containing antibiotics (50 U/ml Penicillin and 50 μg/ml Streptomycin) and 9% (v/v) Fetal Bovine Serum (FBS) (all from Gibco, MD, USA). The C_2_C_12_ cells were plated overnight in GM at 10^4^/cm^2^ on BD Matrigel coated (1:50 in DMEM low glucose) 35 mm dishes as described previously (both from BD Biosciences, MA, USA) [43]. To study effects on myogenesis, differentiation was induced by growth factor withdrawal [44], replacing GM with differentiation medium (DM) (DMEM, low glucose, with 1% heat-inactivated FBS and antibiotics). The synthetic GC dexamethasone (hereinafter referred to as Dex) (Sigma-Aldrich), TNF-α (Calbiochem, CA, USA), with or without LiCl (Sigma-Aldrich) or CHIR99021 (2 μM) (kindly provided by Dr. Cohen, MRC Protein Phosphorylation Unit, University of Dundee, UK) were directly added to the culture medium upon the induction of differentiation and again 24 h later when the cells were provided with fresh DM. The myocytes were allowed to differentiate for a total of 72 h, in absence or presence of Dex (10 μM) or TNF-α (1 ng/ml) prior to analysis of myogenesis markers.

### Myogenic index

As a morphological parameter of myogenesis, the myogenic index was determined to quantitate myoblast fusion. The C_2_C_12_ cells were induced to differentiate for 72 h either in the presence or absence of Dex or TNF-α. After 72 h of differentiation the cells were washed twice in 1× PBS (RT), subsequently fixed in methanol and stained in May-Grünwald Giemsa (Sigma-Aldrich) according to the manufacturer’s instructions. Pictures were taken at 40× and 100× magnifications using an inverted light microscope (Eclipse E800, Nikon, Japan) connected to a digital camera (DXM, 1200 NF, Nikon, Japan). The 100× magnified images were taken in series of four with a fixed overlap. The total number of nuclei in 4 or more fields was counted, and nuclei were assigned to one of three classes: (1) single nucleated myoblasts; (2) dividing or fusing bi-nucleated myoblasts, and (3) multi-nucleated (> 2 nuclei) myotubes. Per condition, 1900 or more nuclei were counted and assigned to either of the above-mentioned classes.

### Stable cell line and luciferase activity determination

Measurements of Troponin I (TnI) promoter activity during differentiation were performed by creating a stable C_2_C_12_ cell line carrying a genomic TnI promoter-luciferase reporter gene as described previously [45]. To determine the luciferase activity, the cells were washed twice in ice-cold 1× PBS, lysed in 1× reporter lysis buffer (Promega, Madison, WI, USA) and stored at −80°C. The lysates were spun at 14000 rpm (4°C) prior to analysis, and the soluble fraction was used to measure the luciferase activity according to the manufacturer’s instructions (Promega). The total protein concentration was assessed using a Bio-Rad protein assay kit (Bio-Rad, CA, USA) according to the manufacturer’s instructions. The data was corrected for total protein content.

### Muscle creatine kinase activity

Myogenic differentiation was assessed biochemically by measuring muscle creatine kinase (MCK) activity. After the induction of differentiation, the C_2_C_12_ cells were washed twice in ice-cold 1× PBS, subsequently lysed in 0.5% Triton X-100, and scraped from the dish with a cell scraper (rubber policeman). The lysates were centrifuged for 2 min at 14000 rpm (4°C), and the supernatant was aliquoted and stored at −80°C to determine the protein content or MCK activity in the presence of 1.25% BSA. The MCK-activity was measured spectrophotometrically (Stanbio Laboratory, TX, USA) [46]. The specific activity was calculated after correction for total protein content [47].

### Western blotting

The muscle tissue was homogenized in ice-cold 1X whole cell lysate buffer (WCL) (50 mM Tris–HCl, pH 7.4; 150 mM NaCl; 1 mM EDTA; 1 mM Na_3_VO_4_; 5 mM NaF; 10% glycerol; 0.5% Nonidet P-40; 1 mM DTT; 1 mM PMSF; 10 μg/ml leupeptin; 1% aprotinin; 10 mM β-glycerophosphate and 1 mM Na-pyro-PO_4_) using a rotating blade tissue homogenizer (Polytron PT 1600E, Kinematica, Switzerland). The C_2_C_12_ cells were washed twice in ice-cold 1× PBS after which they were lysed in 1× reporter lysis buffer and scraped of the dish using cell scrapers. The total protein concentration was assessed by the Thermo Scientific Pierce BCA Protein Assay kit (Pierce Biotechnology, IL, USA) according to the manufacturer’s instructions. The protein lysates were boiled for 5 min at 95°C after addition of 4× Laemmli sample buffer (0.25 M Tris–HCl pH 6.8; 8% (w/v) SDS; 40% (v/v) glycerol; 0.4 M DTT and 0.04% (w/v) Bromophenol Blue). For SDS-PAGE 1–25 μg of protein was loaded per lane and separated on a Criterion XT Precast 4 - 12% Bis-Tris gel (Bio-Rad), followed by transfer to a 0.45 μm Whatman Protran Nitrocellulose Transfer membrane (Whatman GmbH, Germany) by electroblotting (Bio-Rad Criterion Blotter). The nitrocellulose blots were incubated overnight (4°C) with specific antibodies directed against: myosin light chains 1 (MyLC-1) and −3 (MyLC-3) (#F310) (Developmental Studies Hybridoma Bank, Iowa City, IA, USA), myosin heavy chain fast (MyHC-f) (#M4276) (Sigma-Aldrich), p-eIF2Bϵ (Ser^539^) (#44-530G) (Invitrogen), p-mTOR (Ser^2448^) (#2971), mTOR (#2983), p-Akt (Ser^473^) (#9271), Akt (#9272), p-GSK-3β (Ser^9^) (#9336), GSK-3β (#9332), p-p70S6K (Thr^389^) (#9206), p70S6K (#2708), p-4E-BP1 (Thr^37/46^) (#9459), 4E-BP1 (#9452), p-S6 (Ser^235/236^) (#2211), p-FoXO1 (Ser^256^) (#9461), FoXO1 (#2880), p-FoXO3a (Ser^253^) (#9466), FoXO3a (#2497) and GAPDH (#2118) (all from Cell Signaling Technology), diluted in 1× TBS/0.1% Tween-20. The blots were probed with a peroxidase conjugated secondary antibody (#PI-1000) (Vector Laboratories, CA, USA), and visualized using Supersignal WestPico Chemiluminescent Substrate (Pierce Biotechnology) according to the manufacturer’s instructions and exposed to Super RX film (FUJIFILM, Japan). The Western blot films were digitalized using a Bio-Rad GS-800 Densitometer and subsequent quantification was done using Quantity One software (version 4.5.0) (both from Bio-Rad).

### Statistical analysis

The raw data were entered into SPSS (version 20.0) for statistical analysis. All values are represented as means and error bars indicate the standard error of the mean (SEM). Comparisons of mean values were tested parametrically, using a one-way ANOVA followed by a *post hoc* Fischer’s LSD test. The changes in body weight were tested using a mix-model design ANOVA. Mean value comparisons of *in vitro* data were tested non-parametrically, using the Mann–Whitney U-test. A two-tailed probability value (p < 0.05) between groups was considered statistically significant.

## Results

### GSK-3 inhibition prevents pulmonary inflammation-induced skeletal muscle atrophy

**Figure 1:**
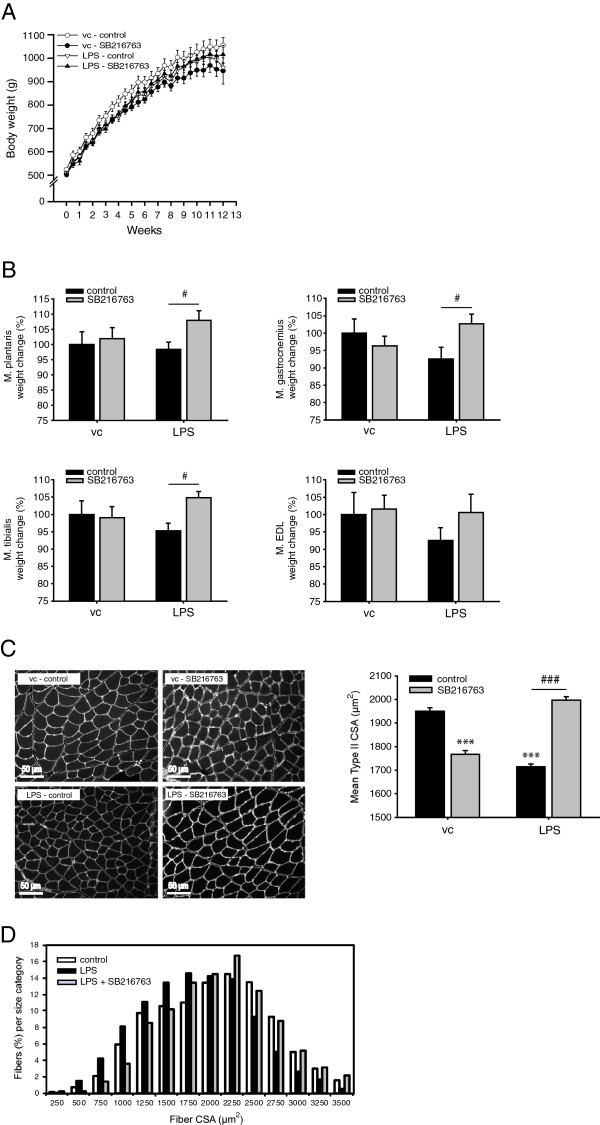
**GSK-3 inhibition prevents skeletal muscle atrophy induced by pulmonary inflammation. (A)** Body weight change of the guinea pigs during the experimental procedures. **(B)** Effects of repeated LPS exposure and GSK-3 inhibition (SB216763) on skeletal muscle wet weights. **(C)** The fiber cross-sectional area (CSA) of muscle fibers in the extensor digitorum longus (EDL) muscle of the guinea pigs was determined from laminin-stained cross-sections. Representative laminin-stained (white) cross-sections of the same region within the EDL muscle for each experimental group (20X magnification, scale bar = 50 μm). Histogram of quantitative analysis of the mean Type II cross-sectional area (CSA) (n = 7 per group). **(D)** Fiber size distribution curves of fiber cross-sectional areas of fibers in the EDL. All data shown represent means ± SEM, n = 9 animals per group. ***p < 0.001 compared with the vc control group; # p < 0.05, ### p < 0.001 refers to a difference between indicated conditions.

Throughout the experimental procedures, neither LPS nor the concomitant administration of LPS and SB216763 significantly affected the increase in body weight of the guinea pigs (Figure 1A). However, from week 4 onwards the increase in body mass of the SB216763-treated saline-challenged group was significantly lower compared with the vehicle-treated, saline-challenged group (p < 0.05) (Figure 1A). Repeated LPS administration consistently appeared to decrease muscle wet weights (M. plantaris: -2%, M. gastrocnemius: -8%, M. tibialis: -5%, M. EDL: -7%), although this did not reach statistical significance (Figure 1B). Intriguingly, SB216763-treatment significantly prevented the LPS-induced reduction in these skeletal muscle weights (except for M. EDL). To verify the effects on muscle mass, the myofiber CSA of the EDL muscle was determined. The glycolytic EDL muscle predominantly consisted of Type II fibers (96.4%, data not shown), and immunohistochemical staining revealed that chronic LPS administration significantly decreased the mean Type II fiber CSA compared with vehicle control-treated muscle (Figure 1C). The decline in Type II fiber CSA following LPS was further substantiated by examining the fiber size distribution curves, which revealed a leftward shift (smaller fiber size) compared with the fiber distribution of vehicle-treated control animals (Figure 1D). Strikingly, pharmacological GSK-3 inhibition abrogated the reduction of mean Type II fiber CSA in response to LPS (Figure 1C and Figure 1D). Unexpectedly, GSK-3 enzyme inhibition caused a significant decrease in mean Type II fiber CSA in EDL muscle of vehicle-treated animals (Figure 1C). Nevertheless, collectively these data indicate that muscle atrophy induced by chronic LPS challenge is prevented by GSK-3 inhibition despite sustained pulmonary inflammation.

### Muscle protein turnover signaling is not affected following chronic LPS-treatment and GSK-3 inhibition

**Figure 2:**
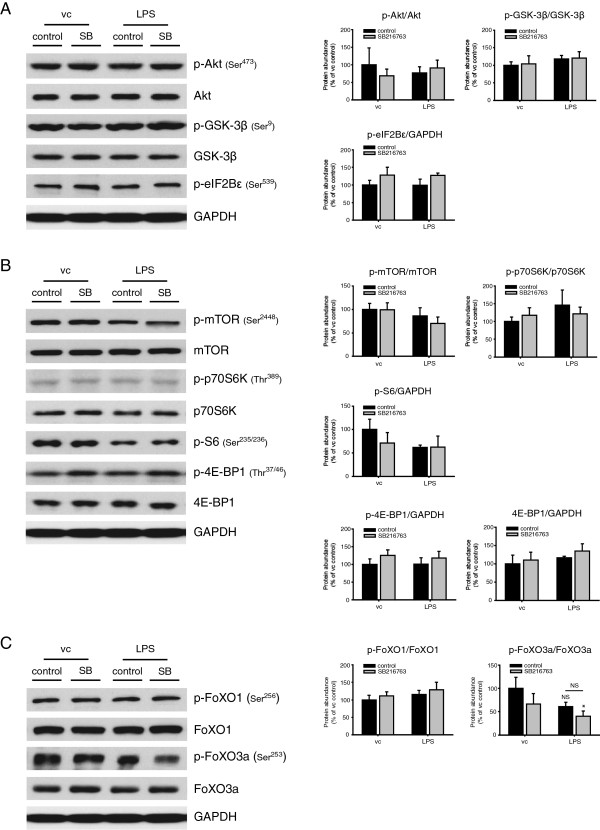
**Muscle protein turnover signaling is not affected following chronic LPS-treatment and GSK-3 inhibition.** Protein synthesis and protein degradation-related signaling molecules were determined in whole muscle homogenates of the extensor digitorum longus (EDL) muscles by Western blot analysis of guinea pigs that were treated intranasally with LPS or SB216763 for 12 weeks. Representative immunoblots depict protein levels of **(A)** phospho-Akt (p-Akt), total Akt, phospho-GSK-3β (p-GSK-3β), total GSK-3β, phospho-eukaryotic initiation factor 2Bϵ (p-eIF2Bϵ), GAPDH, **(B)** phospho-mammalian target of rapamycin (p-mTOR), total mTOR, phospho-p70S6K (p-p70S6K), total p70S6K, phospho-S6 (p-S6), phospho-4E-BP1 (p-4E-BP1), total 4E-BP1, GAPDH, **(C)** phospho-FoXO1 (p-FoXO1), total FoXO1, phospho-FoXO3a (p-FoXO3a), total FoXO3a and GAPDH. The accompanying bar graphs show the densitometric analysis results (means ± SEM, n = 6), represented as a percentage of the vc control group corrected for GAPDH. All data is shown as a ratio of phospho- to total protein for each target (except p-eIF2Bϵ, p-S6 and (p-) 4E-BP1). *p < 0.05 compared with the vc control group. NS: not significant.

To address the potential contribution of altered protein synthesis signaling to the muscle atrophy phenotype, the protein levels and the phosphorylation state of mTOR and its downstream effectors p70S6K and 4E-BP1 as well as Akt, the upstream activator of mTOR were assessed. The phosphorylated (p)-Akt to Akt ratio in LPS control muscle was unchanged following a 12 week treatment regimen with intranasally instilled LPS. Likewise, the p-Akt levels in muscle exposed to SB216763 alone or in combination with LPS remained unaltered, comparable to vehicle/saline-treated controls (Figure 2A). Similarly, the phosphorylation state and abundance of GSK-3β, a direct downstream substrate of Akt, was unaffected in any of the conditions. Chronic pharmacological GSK-3 inhibition by SB216763 in the lung did not result in detectable alterations in the phosphorylation state of the GSK-3β substrate eIF2Bϵ (Figure 2A).

Furthermore, the ratio of p-mTOR over total mTOR was unaffected in any of the conditions. The phosphorylation state of p70S6K, a downstream substrate of mTOR, was unaffected by LPS instillation or GSK-3 inhibition (Figure 2B). In contrast, phosphorylation of S6, a substrate of p70S6K, tended to be reduced upon LPS instillation, but these findings did not reach statistical significance (Figure 2B). Finally, repeated LPS administration or GSK-3 inhibition did not affect p-4E-BP1 or total 4E-BP1 protein abundance, as another downstream substrate of mTOR (Figure 2B). Both phosphorylated levels of FoXO1 as well as total FoXO1 protein abundance remained unaltered following either LPS -or SB216763-treatment (Figure 2C). In contrast, the p-FoXO3a to FoXO3a ratio was reduced in response to concomitant LPS and SB216763-treatment, which is indicative of increased FoXO3a activity (Figure 2C). Altogether these data imply that gross alterations in skeletal muscle protein turnover signaling could not account for the muscle atrophy observed in response to chronic pulmonary inflammation, nor the prevention thereof by pharmacological GSK-3 inhibition.

### GSK-3 inhibition prevents TNF-α-induced impairment of myogenesis

**Figure 3:**
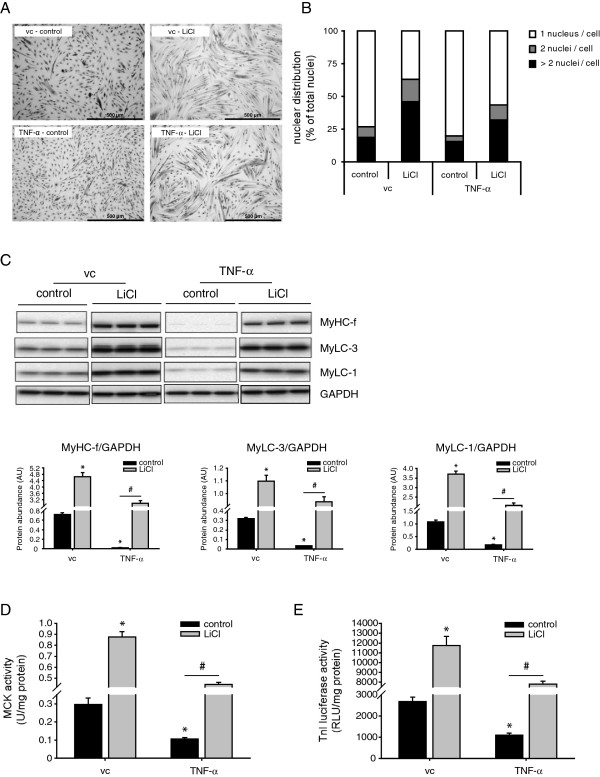
**GSK-3 inhibition by LiCl prevents TNF-α -induced impairment of myogenic differentiation.** Differentiating C_2_C_12_ myoblasts were either cultured in the presence or absence of TNF-α (1 ng/ml), LiCl (10 mM) or vc (1.25% BSA) for 72 h after which the cells were assessed for **(A)** morphological changes by staining with May-Grünwald Giemsa (40X magnification, scale bar = 500 μm). **(B)** Myoblast fusion was quantified by determining the nuclear distribution of 1900 or more nuclei for each separate condition. The data is expressed as a percentage of nuclei residing in cells containing 1, 2, or > 2 nuclei; reflecting mononucleated myoblasts (1 nucleus), dividing or fusing myoblasts (2 nuclei) or myotubes (> 2 nuclei), respectively. **(C)** Next, protein levels of myosin heavy chain fast (MyHC-f), myosin light chains 1 (MyLC-1) and −3 (MyLC-3) and GAPDH were determined in whole cell lysates by Western blot analysis, **(D)** or in cell homogenates muscle creatine kinase (MCK) activity was measured spectrophotometrically, and expressed as specific enzyme activity (units / mg protein). **(E)** Alternatively, differentiating C_2_C_12_ myoblasts containing a stable genomically integrated troponin I (TnI) luciferase reporter construct were cultured for 72 h in the presence or absence of LiCl (10 mM), or TNF-α (1 nM) or vc (1.25% BSA). Subsequently, lysates were prepared to measure luciferase activity (RLU / mg total protein). All data shown are representative of 3 independent experiments (means ± SEM, n = 3). *p < 0.05 compared with vc control; # p < 0.05 refers to a difference between indicated conditions.

In addition to alterations in protein turnover, impaired myogenesis may lie at the basis of sustained muscle wasting [48,49]. Moreover, systemic inflammation resulting from pulmonary inflammation can trigger muscle atrophy [26], and inflammatory cytokines have been shown to contribute to muscle wasting through the inhibition of myogenic differentiation [43]. To investigate whether pharmacological GSK-3 inhibition prevents impaired myogenesis, differentiating C_2_C_12_ myoblasts were cultured in the presence or absence of LiCl and/or TNF-α. LiCl is a direct and indirect inhibitor of GSK-3 and has been widely used to investigate the role of GSK-3 [50,51]. TNF-α supplementation resulted in diminished myogenesis of C_2_C_12_ myocytes (Figure 3A). Subsequent quantification of myotube formation, by determining the myogenic index, clearly demonstrated that TNF-α reduced myoblast fusion (Figure 3B). Conversely, LiCl increased myotube formation, and importantly, markedly attenuated the TNF-α-induced decrease in myotube formation (Figure 3B). TNF-α significantly decreased the myofibrillar protein abundance, i.e. MyHC-f, MyLC-1 and MyLC-3, whereas LiCl stimulated their expression (Figure 3C). Notably, LiCl significantly abrogated the reduction in contractile protein content in response to TNF-α (Figure 3C). In addition to reduced expression of sarcomeric/contractile proteins, TNF-α supplementation markedly decreased MCK activity. Conversely, enzymatic GSK-3 inhibition increased basal MCK activity and prevented the TNF-α-induced decline in MCK activity (Figure 3D). The differentiation-induced transcriptional activation of the TnI promoter was diminished in response to TNF-α, and increased following GSK-3 inhibition (Figure 3E). In line with the other markers of myogenesis, LiCl-treatment significantly reversed the reduction in TnI promoter transactivation in response to TNF-α.

### GSK-3 inhibition blocks glucocorticoid-induced inhibition of myogenesis

**Figure 4:**
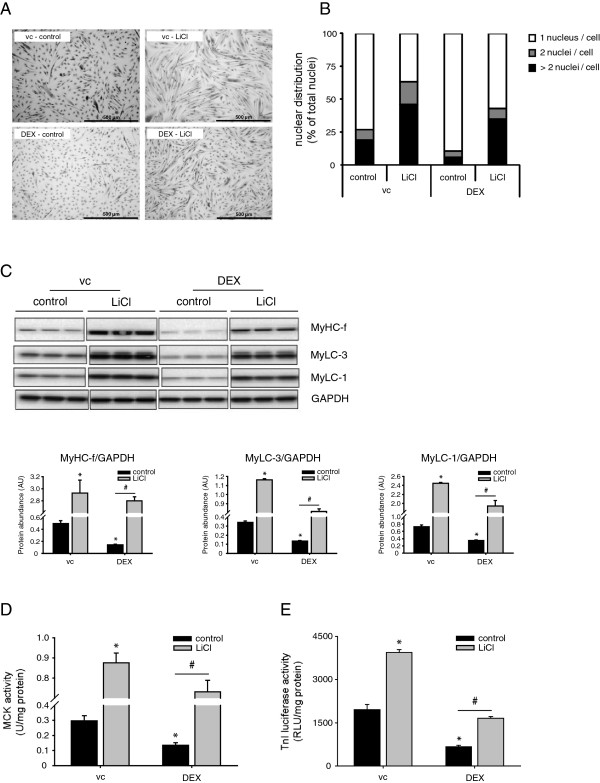
**GSK-3 inhibition by LiCl blocks glucocorticoid-induced inhibition of myogenesis.** Differentiating C_2_C_12_ myoblasts were either cultured in the presence or absence of dexamethasone (Dex) (10 μM), LiCl (10 mM) or vc (DMSO) for 72 h after which the cells were assessed for **(A)** morphological changes by staining with May-Grünwald Giemsa (40× magnification, scale bar = 500 μm). **(B)** Myoblast fusion was quantified by determining the nuclear distribution of 1900 or more nuclei for each separate condition. The data is expressed as a percentage of nuclei residing in cells containing 1, 2, or > 2 nuclei; reflecting mononucleated myoblasts (1 nucleus), dividing or fusing myoblasts (2 nuclei) or myotubes (> 2 nuclei), respectively. **(C)** Next, protein levels of MyHC-f, MyLC-1, MyLC-3 and GAPDH were determined in whole cell lysates by Western blot analysis, **(D)** and MCK activity was measured spectrophotometrically, expressed as specific enzyme activity (units / mg protein). **(E)** Alternatively, differentiating C_2_C_12_ myoblasts containing a stable genomically integrated troponin I (TnI) luciferase reporter construct were cultured for 72 h in the presence or absence of LiCl (10 mM), or Dex (10 μM) or vc (DMSO). Subsequently, lysates were prepared to measure luciferase activity (RLU/mg total protein). All data shown are representative of 3 independent experiments (means ± SEM, n = 3). *p < 0.05 compared with vc control; # p < 0.05 refers to a difference between indicated conditions.

**Figure 5:**
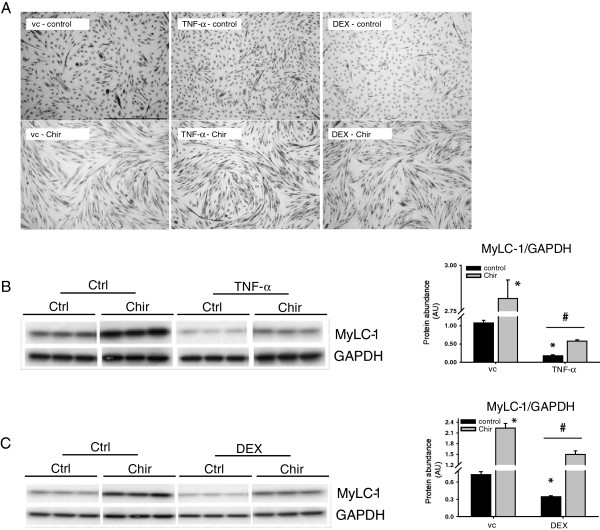
**GSK-3 inhibition using CHIR99021 prevents TNF-α- or glucocorticoid-induced inhibition of myogenesis.** Differentiating C_2_C_12_ myoblasts were either cultured in the presence or absence of TNF-α (1 ng/ml), dexamethasone (Dex) (10 μM), LiCl (10 mM) or CHIR99021 (2 μM) or vc (DMSO) for 72 h after which the cells were assessed for **(A)** morphological changes by staining with May-Grünwald Giemsa (40X magnification, scale bar = 500 μm). **(B, C)** Next, protein levels of MyLC-1 and GAPDH were determined in whole cell lysates by Western blot analysis. All data shown are representative of 3 independent experiments (means ± SEM, n = 3). *p < 0.05 compared with vc control; # p < 0.05 refers to a difference between indicated conditions.

Systemic inflammation increases circulating levels of cortisol; a potent trigger of muscle atrophy [52,53]. Repeated intranasal LPS instillation in guinea pigs resulted in an increase in plasma cortisol levels (229%, ± 48.4%), which was unaffected by SB213763-treatment (172%, ±SEM 51.2%). Previously it was demonstrated that the synthetic GCs prednisolone as well as Dex strongly impair myogenesis [54]. The addition of Dex to the culture medium during differentiation resulted in impaired C_2_C_12_ myotube formation (Figure 4A). Similar to the results obtained with TNF-α, pharmacological GSK-3 significantly prevented impairment of myoblast fusion in the presence of Dex (Figure 4B). Furthermore, Dex significantly decreased the muscle-specific protein expression of MyHC-f, MyLC-1 and MyLC-3, while LiCl supplementation completely prevented this effect (Figure 4C). Moreover, Dex markedly reduced MCK activity (Figure 4D) and TnI promoter transactivation (Figure 4E), which was prevented in the presence of LiCl (Figure 4D and Figure 4E, respectively). To ascribe the preventive effects of LiCl on impaired myogenic differentiation by TNF-alpha or Dex to inhibition of GSK-3 enzymatic activity, the structurally unrelated GSK-3 inhibitor CHIR99021 was deployed. Incubation of differentiating myoblasts with CHIR99021 prevented or attenuated TNF-alpha-induced blockade of myogenic fusion or MyLC accumulation (Figure 5A, B), similar as observed with LiCl. Likewise, pharmacological GSK-3 inhibition using CHIR99021 reversed the Dex-induced impairment of myogenesis (Figure 5A, C).

## Discussion

Pulmonary and systemic inflammation in COPD has been associated with several extra-pulmonary consequences of the disease [55,56]. A prominent systemic manifestation of COPD is skeletal muscle atrophy [5,24,25], and the results presented in this manuscript demonstrate that pharmacological GSK-3 inhibition is beneficial in preventing muscle wasting in a model of chronic pulmonary inflammation, without affecting pulmonary inflammation per se as shown in the companion paper of this manuscript [57]. Further, impaired myogenic differentiation of cultured muscle cells, in response to TNF-α and GCs as putative mediators of systemic inflammation-induced muscle atrophy, was restored by GSK-3 inhibition, putting forward sustained myogenesis as a potential basis for the maintenance of muscle mass despite pulmonary inflammation observed in this study.

Pulmonary inflammation was induced by repeated intranasal instillation of LPS, an endotoxin that has been associated with the development of COPD [1,58,59]. Interestingly, the data presented in the companion paper revealed that pulmonary inflammation was not affected by GSK-3 inhibition [57] suggest that any effects of local SB216763 instillation on systemic pathology are not accounted for by alterations in the lung inflammatory response. Chronic LPS-treatment resulted in skeletal muscle atrophy. Similarly, previous work by our group showed that acute pulmonary inflammation was associated with muscle atrophy following intra-tracheal LPS instillation (IT-LPS) [26]. In that study, local inflammation was accompanied by a potent systemic inflammatory response, characterized by elevated circulating levels of inflammatory cytokines, which coincided with increased NF-κB signaling in skeletal muscle [26]. Systemic inflammation has been shown to contribute significantly to skeletal muscle atrophy and pro-inflammatory cytokines have been suggested to induce and mediate catabolic responses in muscle via NF-κB signaling [26,60]. In the current study circulating cytokine levels were not assessed, rendering it difficult to implicate systemic inflammation as a direct causal trigger in the onset of muscle atrophy. Nevertheless, it is conceivable that, considering the persistent inflammatory state of the lung, systemic inflammation was sustained following repeated LPS challenge, as increased circulating levels of inflammatory cytokines were reported in a mouse model of chronic pulmonary inflammation [24].

During the early onset of inflammation, TNF-α and IL-1β stimulate the release of GCs, as an endogenous reaction to dampen the inflammatory response, via activation of the hypothalamic-pituitary-adrenal (HPA) axis [52]. In this study, pulmonary inflammation was associated with increases in plasma cortisol levels, providing indirect evidence to support the notion that systemic inflammation might have occurred in this model. Previously, IT-LPS instillation was reported to increase the plasma concentration of corticosterone; the endogenous GC in mice [61], and in other models of inflammation -or GC-associated muscle atrophy administration of GR-receptor antagonists prevented or attenuated muscle atrophy [62,63].

Indeed, LPS-induced increases in plasma cortisol were paralleled by a significant decrease in myofiber CSA, and only the latter was prevented by GSK-3 inhibition. Remarkably, basal GSK-3 inhibition likewise resulted in a decrease in myofiber CSA, which may have been the consequence of a blunted increase in body weight in response to SB216763. Over-inhibition of GSK-3 under normal circumstances may not be favorable as GSK-3 is essential in the control of various physiological processes such as development and cell proliferation [64,65]. As elevated GSK-3 activity was previously reported in atrophying muscle [66], our data may indicate that the use of GSK-3 inhibitors should be limited to conditions characterized by aberrant GSK-3 regulation, aimed at restoration of physiological GSK-3 activity levels [65,67]. Nevertheless, pharmacological GSK-3 inhibition resulted in significant sparing of muscle mass and myofiber CSA, despite sustained pulmonary inflammation and elevated cortisol levels. This is in line with previously reported studies highlighting the efficacy of GSK-3 inhibitors in reducing proteolysis in septic muscle [68], and in muscles from burned rats [69]. Furthermore, GSK-3 inhibition was demonstrated to decrease general protein degradation comparably to IGF-I in a model of GC-induced muscle proteolysis [70], and earlier work by our lab delineated a pivotal role for GSK-3β in the induction of skeletal muscle atrophy, as loss of GSK-3β expression in muscle resulted in specific sparing of myofibrillar protein abundance following synthetic GC-treatment [40]. Thus, the inability of GSK-3 inhibition to reduce pulmonary inflammation implies that the SB216763 inhibitor may have directly inhibited GSK-3 in muscle.

In view of the significance of GSK-3 signaling in the processes that determine muscle mass [40,65], markers of protein synthesis and degradation were assessed in muscle homogenates. As indicated earlier, Akt activation results in the phosphorylation and cytoplasmic retention of the FoXO transcription factors, and is responsible for the subsequent attenuation of protein breakdown. Conversely, reduced phosphorylation of FoXO, consequent to diminished Akt activity, may increase proteolysis signaling, and hence muscle atrophy. Yet, pulmonary inflammation only appeared to marginally reduce p-FoXO3a protein levels, while the phosphorylation status of FoXO1 remained unaffected. It is noteworthy that suppression of GSK-3 activity did not influence the phosphorylation of FoXO under any conditions. Of note, these moderate effects of pulmonary inflammation and GSK-3 inhibition on FoXO corresponded to the unaltered phosphorylation state of Akt; its upstream regulator. Subsequent analysis of other protein synthesis signaling markers, downstream of Akt, revealed no demonstrable effects of either LPS -or SB216763-treatment. In contrast, several *in vivo* studies established that LPS-treatment resulted in suppressed protein synthesis in muscle [71,72]. However, these inhibitory effects on protein synthesis were measured in the acute phase, and a recent report by Tarabees *et al.* suggested that endotoxins only transiently decrease protein synthesis in skeletal muscle through Akt [73].

A limitation of this study was the fact that besides FoXO, no additional analyses on muscle protein breakdown signaling were included. Acute loss of muscle mass typically involves increased proteolysis, in which an important contribution of the ubiquitin 26S-proteasome system (UPS), and largely depends on the rate-limiting E3 ubiquitin ligases atrogin-1 (MAFbx) and muscle RING finger 1 (MuRF1) has been postulated [13,74,75]. Due to limited reagent availability these targets could not be measured in guinea pig muscle. Although our findings are not in support of a major role of altered protein turnover in the development of muscle atrophy in this chronic model, it is not possible to conclusively rule out its contribution. First of all, no actual measurements of muscle protein synthesis and degradation were conducted, and the signaling cues of protein turnover, as assessed here, may not always correspond with changes in protein synthesis and degradation [76,77]. Secondly, increased proteolysis has been reported in response to acute pulmonary inflammation [26,78]. As elevated muscle breakdown signaling requires GSK-3β activity [40,68], SB216763-treatment may have prevented an initial decrease in muscle mass, which subsequently did not recover in the LPS control group despite the normalization of proteolysis signaling.

Nevertheless, our data suggested that the sustained muscle atrophy phenotype was not the consequence of gross alterations in protein turnover. Besides protein turnover, myonuclear turnover constitutes another cellular mechanism determining muscle mass [17]. The sustained nature of the muscle atrophy phenotype, may have involved impaired regeneration following recovery from atrophy, resulting in impaired restoration of muscle mass. Intriguingly, pharmacological and physiological GSK-3 inhibition was recently shown to enhance myoblast fusion and myotube formation, ascribing an important role to GSK-3 in the process of myogenesis [41,79]. In the present study, we made use of the C_2_C_12_ cell culture model to investigate whether GSK-3 inhibition could prevent impaired myogenesis in response to TNF-α and the synthetic GC Dex. Impaired myogenic differentiation in response to TNF-α [80] has been reported previously, and several lines of evidence, including our own work, have demonstrated that, besides their well-described role as inducers of muscle proteolysis, GCs can also cause muscle atrophy by inhibiting several aspects of myogenesis [54,81,82].

In agreement with previous findings, TNF-α significantly impaired myogenesis in cultured muscle cells, whereas GSK-3 inhibition improved myogenic differentiation. Importantly, pharmacological GSK-3 inhibition, using two structurally unrelated inhibitors, completely prevented reduced myogenesis in response to TNF-α. Similarly, the Dex-induced impairment of myogenesis was completely blocked by GSK-3 inhibition using either LiCl or CHIR99021. Taken together, interference with myogenic differentiation, as a direct consequence of circulating inflammatory mediators or secondary to increased GC levels, may have resulted in myofiber atrophy by impaired myogenesis, whereas this process was sustained by GSK-3 inhibition, resulting in preservation of muscle mass.

Collectively, our data demonstrates that topical application of the selective GSK-3 inhibitor SB216763 is capable of preventing skeletal muscle atrophy in a guinea pig model of pulmonary inflammation. These findings warrant further exploration of pharmacological inhibition of GSK-3 as a novel therapeutic strategy in the treatment of COPD-associated skeletal muscle wasting.

## Competing interests

The authors declare that they have no competing interests.

## Authors’ contributions

KV carried out the muscle dissections, data interpretation and conception of the manuscript, HB was responsible for experimental design, execution of the experimental protocol and involved in the conception of the manuscript, NP conducted and interpreted the *in vitro* studies, AR and AH were responsible for setting up and carrying out muscle dissections and tissue processing, AS was involved in the conception of the manuscript, RG was responsible for conception of the study and manuscript, RL was responsible for the data interpretation and conception of the manuscript. All authors have read and approved the final manuscript.
